# Exploring the impact of acute solvent exposure on larval zebrafish behaviour

**DOI:** 10.3389/fnbeh.2025.1717998

**Published:** 2025-11-28

**Authors:** Ethan V. Hagen, Matthew M. M. Harper, Yanbo Zhang, Trevor J. Hamilton

**Affiliations:** 1 Department of Psychiatry, University of Alberta, Edmonton, AB, Canada; 2 Department of Psychology, MacEwan University, Edmonton, AB, Canada; 3 Neuroscience and Mental Health Institute, University of Alberta, Edmonton, AB, Canada; 4 Allen Discovery Center for Neurobiology in Changing Environments, University of California, San Diego, San Diego, CA, United States

**Keywords:** pharmacology, toxicology, *Danio rerio*, DMSO, methanol, ethanol, startle responses

## Abstract

Zebrafish (*Danio rerio*) are commonly used to test the impact of pharmacological and toxicological compounds. Larval zebrafish are extensively used because of high throughput procedures allowing simultaneous behavioural measurement in 24-, 48-, or 96-well plates. Often solvents are used as a vehicle for poorly soluble or insoluble compounds, however, the impact of dimethyl sulfoxide (DMSO), methanol, and ethanol after acute administration is not well characterized. Here we investigated the impact of 30-min exposures of DMSO, methanol, and ethanol (0.01%, 0.1%, and 1.0% vol/vol) on 5-day old larval zebrafish locomotion and startle responses. We found no effect of DMSO on distance moved and thigmotaxis in a spontaneous swimming test, and no effect on dark-, light-, or tap-startle responses compared to controls. Methanol and ethanol, both at 1.0% increased the distance moved, and ethanol decreased the dark startle response at 1.0%. Neither ethanol nor methanol had any impact on time in thigmotaxis zone, light- or tap-startle responses. Results from this study suggest that with acute exposure to experimental compounds requiring a solvent, the least impact on behaviour would occur with DMSO, followed by methanol, then ethanol.

## Introduction

The use of zebrafish (*Danio rerio*) as a model organism is becoming foundational for advances in medicine, pharmacology, and toxicology. They can be used at every developmental stage (embryonic, larval, juvenile, and adult) to address an array of scientific questions. The embryonic stage occurs from fertilization until hatching around 2–3 days post-fertilization (dpf), when they enter the larval stage, which proceeds to 4–6 weeks of life (Singleman and Holtzman, 2014). Zebrafish progress from the juvenile stage to adulthood at 3 months when they are capable of breeding; a relatively rapid breeding cycle compared to mammals, and notably zebrafish females can produce multiple batches of eggs (clutches) within a short period of time (Tavares and Santos Lopes, 2013). Zebrafish use external fertilization which is valuable in long term behavioural studies due to lack of paternal effects during development (Nasiadka and Clark, 2012). Larval zebrafish are transparent, allowing internal organs to be monitored throughout their development (Goessling and Sadler, 2015) and their brain activity can be captured via real-time *in vivo* imaging experiments (Lovett-Barron, 2021). Larval zebrafish are capable of a range of behaviours starting at 72 h post fertilization (hpf) including response to light or dark, acoustic startle, and response to novel environments (Ahmad et al., 2012). Larval zebrafish show distinct swim patterns in light and dark exposures after 4 dpf (Basnet et al., 2019; Burgess and Granato, 2007a) and have shown behavioural responses to mechanical startle/tapping stimuli (Burgess and Granato, 2007b). Larvae at 5 dpf demonstrate more complex behaviour, exhibiting responses to visual and acoustic stimuli (Fero et al., 2011).

Larval zebrafish are widely used for high-throughput drug and toxin screening due to their rapid development, small size, transparent embryos, and efficiency of behavioural tracking systems, together allowing for high-throughput generation of data (Yang et al., 2018; Cassar et al., 2017). Depending on the scientific question, larval zebrafish can be used in developmental experiments, with acute or chronic exposure to pharmacological or toxicological compounds. Larval zebrafish regulate ions and small molecules via passive diffusion through their skin (Glover et al., 2013; Perry et al., 2023) with gills beginning to function around 5 dpf (van Wijk et al., 2019; Thiruppathy et al., 2022). With both of these mechanisms, at 5 dpf, the larval zebrafish is capable of absorbing compounds of interest rapidly and reliably via immersion (Cafora et al., 2024; Matsui et al., 2006; van Wijk et al., 2019). Small molecules like ethanol, methanol, and dimethyl sulfoxide (DMSO) are readily absorbed and distributed throughout the circulatory system, with ethanol and methanol eventually metabolized by alcohol dehydrogenase (Tsedensodnom et al., 2013). The metabolism and excretion of DMSO is less understood in fish and may get excreted without any breakdown. Many screening methods for pharmaceuticals and toxins use acute exposures around 30 min, followed by recording of larval behavioural and motor responses to examine behavioural and nervous system changes. For example, Yang et al. (2018) developed an automated photomotor response assay exposing 7 dpf larvae to hypnotic/sedative drugs for approximately 30 minutes. Similarly, using a light/dark behavioural assay, Jarema et al. (2022) exposed larvae acutely for 30 min to psychedelics and other compounds (Cassar et al., 2017). These studies demonstrate that short-duration exposures are effective in detecting rapid behavioural and physiological responses. Moving forward, it is practical to examine the impact of 30-min exposures to the solvents most often used along with the administration of lipophilic compounds.

Solvents are commonly used in zebrafish experiments to dissolve and deliver compounds that are not soluble in water. They allow for compound stability, and delivery consistency, especially with experiments dosing fish in the compound of interest (i.e., water immersion). Many solvents are available, with DMSO, methanol, and ethanol being three of the most commonly used. DMSO is an effective solvent used frequently in zebrafish research (Hallare et al., 2004; Steenbergen et al., 2011; Kyzar et al., 2012; Scatterty and Hamilton, 2024; Johnson et al., 2023; de Koning et al., 2015) due to its ability to dissolve a broad range of both polar and nonpolar organic and inorganic compounds (Martin et al., 1967). However, DMSO does not solubilize all chemicals effectively, necessitating the use of other solvents like ethanol or methanol (Maes et al., 2012; López Patiño et al., 2008; Johnson et al., 2023; Amin et al., 2025; Hamilton et al., 2021). The use of solvents like DMSO, ethanol, and methanol are crucial when dosing larval zebrafish with lipophilic chemicals to maintain the high-throughput nature of experiments (Chen et al., 2011; Adefolaju et al., 2015). However, despite their utility, these solvents can independently induce behavioural and developmental alterations in larval zebrafish. For example, chronic exposure to ethanol and DMSO can modify locomotor activity without causing gross morphological defects (Chen et al., 2011). Similar behavioural effects have been observed in other aquatic organisms, such as *Daphnia magna* exposed to DMSO (Huang et al., 2018). It is therefore critical to carefully control solvent doses and exposure durations to mitigate confounding effects on larval zebrafish behaviour and development (Maes et al., 2012). Recent investigations have highlighted the dose-dependent physiological and morphological changes induced by DMSO at doses exceeding 1% (Gomes et al., 2025; Hoyberghs et al., 2021). There have been large scale studies on the impact of solvents on zebrafish development after chronic exposure (Hoyberghs et al., 2021; Chen et al., 2011; Hedge et al., 2023; Xiong et al., 2017), however, there are few studies examining the sub-lethal impact of acute exposure on larval zebrafish locomotion and startle responses.

Ethanol, methanol, and DMSO are all small molecules that can readily enter the central nervous system (CNS) of zebrafish. In the brain, ethanol interacts with a variety of neurotransmitter systems along various pathways (Levinthal and Hamilton, 2022). In zebrafish, ethanol interacts with dopamine, serotonin, GABA, aspartate, glycine, taurine (Chatterjee and Gerlai, 2009; Facciol and Gerlai, 2020; Goodman and Wong, 2020; Bhandari et al., 2024) and acetylcholine (Rico et al., 2007) systems. The interaction of methanol with neurochemicals is less well studied, however it causes CNS depression (Alrashed et al., 2024), likely through similar mechanisms as ethanol, and its metabolites account for its toxicity with the retina being highly sensitive (Ashurst et al., 2025). In larval zebrafish methanol inhibits acetylcholinesterase activity (Rico et al., 2006) and alters retinal structure and function (Fu et al., 2017). The mechanism of DMSO in the zebrafish has yet to be studied, but at doses ≥0.1% there is an increase in chorion permeability (Kais et al., 2013).

In this study we investigated the behavioural effects of acute (30-min) exposure to DMSO, methanol, and ethanol at doses of 0%, 0.01%, 0.1%, or 1.0% vol/vol on larval zebrafish (5 dpf) by assessing their spontaneous locomotion, visual and mechanical startle responses. Behavioural variables of interest included distance moved and time spent in the thigmotaxis zone during a spontaneous swimming test, and startle responses to dark, light, and a mechanical tap.

## Materials and methods

### Animal and housing

Adult zebrafish were housed in 10 L polycarbonate tanks in a Tecniplast ZebTEC multilinking habitat system (Tecniplast Group, Toronto, ON, Canada). Zebrafish used were a MacEwan-bred hybrid strain (third-generation wildtype × AB cross), with the AB strain originating from Dalhousie University (Halifax, NS, Canada), and the original hybrid strain originating from the University of Ottawa (Ottawa, ON, Canada). The system continuously circulated and filtered the habitat water through 100% polyester pleated mechanical filters and 5 μm activated carbon filters and then under UV light. Automatic water changes occurred via a 5-step filtration process, paired with non-iodized salt, sodium bicarbonate and acetic acid buffering. The pH was maintained between 6.5 and 8.0, while the water temperature was set to 28.5 °C ± 1 °C and conductivity was set to 1,000 μS. Fish were fed Gemma Micro 300 fish flakes (Skretting, Tooele, UT, USA) twice daily (am/pm). In the habitat room an automated 14-h light/dark cycle was adhered to (7:00 to 21:00) with an ambient room temperature of 27 °C. Daily husbandry and water quality tasks were completed by a MacEwan University Animal Care Coordinator.

Larval zebrafish (*n* = 288) were bred in-house using hybrid zebrafish, as described above. Breeding procedures began by placing two males and three females into a separate breeding tank within the habitat system. Sex was determined via visual inspection (Hagen et al., 2025; Hagen et al., 2024; Darrow and Harris, 2004; Spence and Smith, 2007). Breeding tanks (3 L) contained a sloped breeding insert inside with a clear dividing wall. Small green artificial plants were placed into the breeding tanks for environmental enrichment. Additional visual enrichment was provided via wrapping around the breeding tank which contained images of rocks and plants. Fish were chosen from the same age cohort (~8 months) and breeding pairs were placed in the tanks situated on an adjacent shelf around 16:00. The following morning, around 9:00, the clear dividing wall was removed allowing the fish to spawn. Fish were allowed ~30-min to spawn before the eggs were collected. Following egg collection, the breeding adults were returned to their home tank and fed. The fertilized eggs were transferred into a petri dish and cleaned. Following the cleaning the eggs were placed into zebrafish embryo media which was made by adding 20 mL of a 50X E3 solution (NaCl: 7.3 g, KCl 0.325 g, CaCL: 1.1 g, MgSO_4_: 2.025 g, dH_2_O: 500 mL) to 1 L dH_2_O and 200 μL of 0.05% methylene blue to make the 1x zebrafish embryo media, resulting in a 0.00005% concentration of methylene blue, which is within the Canadian Council on Animal Care guidelines (CCAC, 2020) (although methylene blue has recently been found to alter oxidative energy metabolism (Nipu et al., 2025), it does not seem to alter development or behaviour (Hedge et al., 2023)). Following the addition of the embryonic zebrafish medium, embryos were placed in a Tritech Research DigiTherm incubator (Tritech Research, Inc., CA, USA) which maintained the same light/dark schedule as the habitat room, and with an internal temperature of 28.5 °C. Each day the embryo media was changed, and unviable embryos were removed using a micropipette. Embryo viability was checked visually via microscopy (Fisher scientific Inc., NH, USA; Olympus Canada Inc., ON, Canada). Experiments were performed under MacEwan University Animal Ethics Board (AREB) under protocol number 101853 in compliance with the Canadian Council on Animal Care (CCAC) experimental guidelines, and were carried out in compliance with ARRIVE guidelines for animal research.

### Dosing

**Figure 1:**
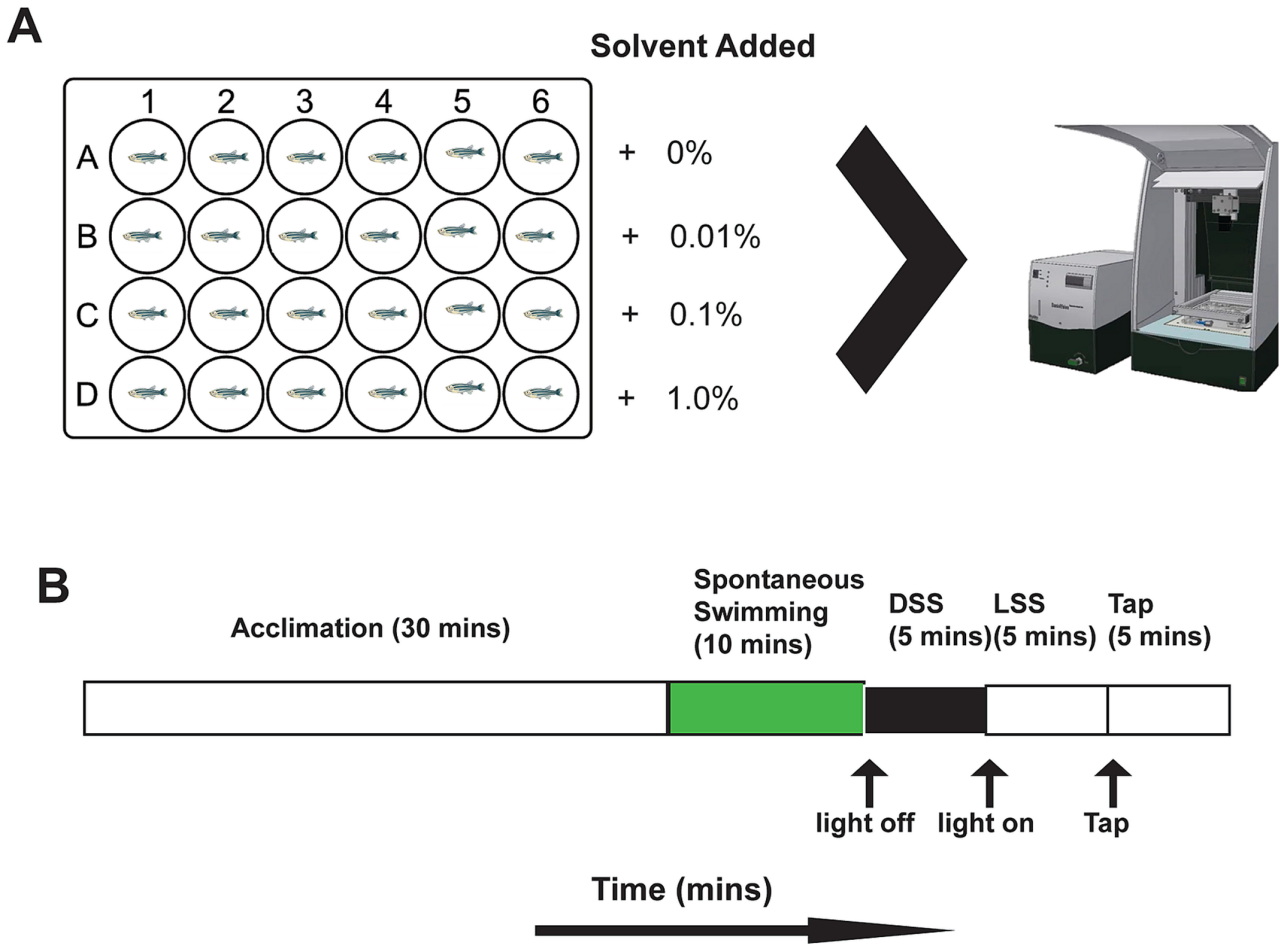
Experimental setup. **(A)** 24-well plate setup showing cartoon zebrafish in each well. Compounds were administered in rows and were randomly allocated for each well plate. When larval fish were placed in the wells they were moved to DanioVision for motion-tracking. Image from noldus.com. **(B)** Timeline of behaviour testing in the DanioVision system. The protocol began with 30 min of acclimation with the internal lights on. Recording then occurred for 10 min of spontaneous swimming with the lights on. Next, the lights were turned off for 5 min and the “dark startle response” (DSS) was recorded. After 5 min of darkness the lights were turned back on to record the “light startle response” (LSS). After 5 min the mechanical tap occurred (TSS) with the lights on.

The timing of experimental procedures, including exposure to solvents, were standardized over each testing day. Compound solutions were prepared at twice the desired final concentration (2X) and subsequently diluted to the target concentration using zebrafish embryo medium directly in the well plates. The solvents utilized included dimethyl sulfoxide (DMSO; CAS-No: 67-68-5) (Sigma, ON, Canada), ethanol (CAS-No: 64-17-5) (Greenfield, ON, Canada) and methanol (CAS-No: 67-56-1) (Sigma, ON, Canada). Each stock solution was thoroughly mixed by shaking prior to aliquoting into wells. To achieve the intended dilution, 900 μL of the 2X solvent solution was added to 900 μL zebrafish embryo medium in each well. For each dose and solvent condition, 24 larvae, from the available clutches, were assigned per group (total *n* = 288). Only one solvent was tested per day. Solvent doses examined were 0.0, 0.01, 0.1, and 1.0% vol/vol (Figure 1A). Larvae were sourced from multiple clutches over the course of testing. For each experimental run, the designated dose row was rotated between plates to mitigate potential directional biases. On the day of testing (5 dpf), all larvae were transferred from their housing petri dishes to the testing plates containing the solvents. All data collection and subsequent analyses were performed with researchers blinded to experimental conditions.

### Behavioural testing

On the day of testing, larvae were visually inspected under a microscope and assessed for responsiveness through gentle physical manipulation during pipetting to confirm viability. Any larvae determined to be deceased were recorded and excluded from subsequent motion tracking analyses. Mortality was observed after testing in larvae exposed to ethanol at doses of 0.01% (*n* = 2), 0.1% (*n* = 2), and 1.0% (*n* = 1) and these larvae were removed from the study. There was no lethality in any other group.

Behavioural assays were conducted using a Noldus DanioVision system coupled with EthoVision XT 17 software (Noldus Information Technology, Wageningen, The Netherlands) for automated tracking of 5 dpf larvae and delivery of startle response stimuli. All videos were captured at 30 frames per second. The 24-well plates were populated with 24 larvae per plate, comprising six larvae from each solvent dosage group and six controls. Plates were placed in the DanioVision chamber for a 30-min acclimation period with interior lighting on (100% luminosity; 10,000 lux); behaviour was recorded but not actively tracked during acclimation. Following acclimation, spontaneous swimming behaviour was recorded under constant light conditions for 10-min, focusing on total distance moved (mm) and time spent in the thigmotaxis zone (seconds). The arena (16.2 mm diameter) was divided in two by a virtual circle placed in the center of the arena (8.1 mm diameter) with the outer zone from the circle to the arena wall being the thigmotaxis zone. Immediately thereafter, larvae underwent a dark startle stimulus (DSS), which involved a sudden transition from light to darkness; behavioural responses were analyzed by comparing distance moved during the 5 s before and 5 s after the light-off event. The dark phase continued for an additional 5-min. Subsequently, a light startle stimulus (LSS) entailed a sudden transition from dark to fully illuminated conditions (100% luminosity) with similar analyses comparing movement 5 s before and after light onset, followed by a 5-min light recovery period. Finally, a tapping startle stimulus (TSS) was administered, consisting of a single maximal intensity tap (intensity: 8). The primary behavioural focus for the TSS was the response to the tap, quantified by comparing distance moved during the 5 s pre- and post-tap (Figures 1A,B). This structured protocol allowed comprehensive evaluation of larval behavioural responses to acute sensory stimuli under varying solvent exposures. Data interpolation was applied in EthoVision to account for any missing frames during motion tracking.

### Statistical analysis

All statistical analyses were performed using GraphPad Prism version 10 (GraphPad Software, San Diego, CA). Normality of the data was assessed using the D’Agostino-Pearson test. Since all datasets deviated from normality, nonparametric analyses were conducted using the Kruskal–Wallis test followed by Dunn’s multiple comparisons *post-hoc* test. The significance threshold was set at *α* = 0.05 for all analyses. Startle responses were quantified as the difference between post-stimulus and pre-stimulus values (distance moved during 5 s after stimulus minus distance moved during 5 s before stimulus). No outliers or data points were excluded from the analyses.

## Results

In this experiment we tested the impact of three commonly used solvents, ethanol, methanol, and DMSO (0, 0.01, 0.1%, or 1.0% vol/vol; *n* = 22–24 per group) in larval zebrafish (5 dpf). We quantified distance moved and time near the walls (thigmotaxis) in a spontaneous movement test, then measured three startle responses caused by a sudden shift to darkness (DSS), sudden shift to light (LSS), and response to a mechanical tap stimuli (TSS).

### DMSO

**Figure 2:**
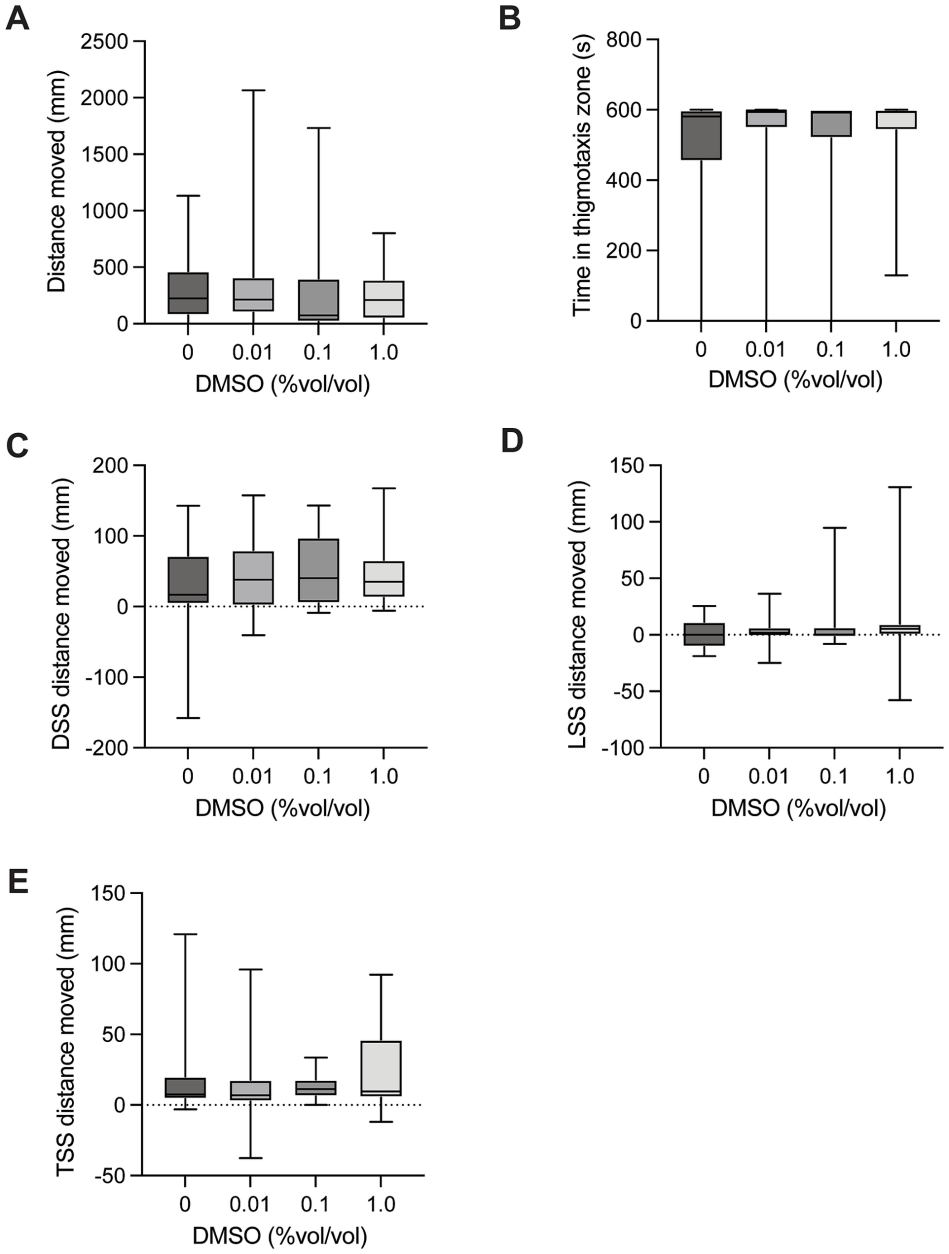
Behavioural responses to DMSO dosing. **(A)** Total distance moved in the 10-min spontaneous swim test. **(B)** Total time in the outer thigmotaxis zone during the spontaneous swim test. **(C)** Change in distance moved following the dark startle stimuli (5 s after DSS – 5 s before DSS). **(D)** Change in distance moved following the light startle stimuli (5 s after LSS – 5 s before LSS). **(E)** Change in distance moved following the tap startle stimuli (5 s after 1st TSS – 5 s before 1st TSS). Boxes represent the 25th and 75th percentiles, and whiskers represent the smallest and largest values. The line in the boxes is plotted at the median.

There was no significant difference in distance moved between control larvae and groups exposed to DMSO [H(3) = 1.999, *p* = 0.5726, Figure 2A] in the spontaneous movement test. There was no significant difference in time spent in the thigmotaxis zone between control larvae and groups exposed to DMSO [H(3) = 1.832, *p* = 0.6080, Figure 2B] when testing spontaneous movement. There was no significant difference in dark startle response (5 s after light off – 5 s before light off) on distance moved in control larvae and groups exposed to DMSO [H(3) = 0.8327, *p* = 0.8700, Figure 2C]. There was no significant difference in light startle response (5 s after light on – 5 s before light on) on distance moved in control larvae and groups exposed to DMSO [H(3) = 3.826, *p* = 0.2809, Figure 2D]. There was no significant difference in mechanical tap startle response (5 s after tap – 5 s before tap) on distance moved in control larvae and groups exposed to DMSO [H(3) = 2.193, *p* = 0.5333, Figure 2E].

### Methanol

**Figure 3:**
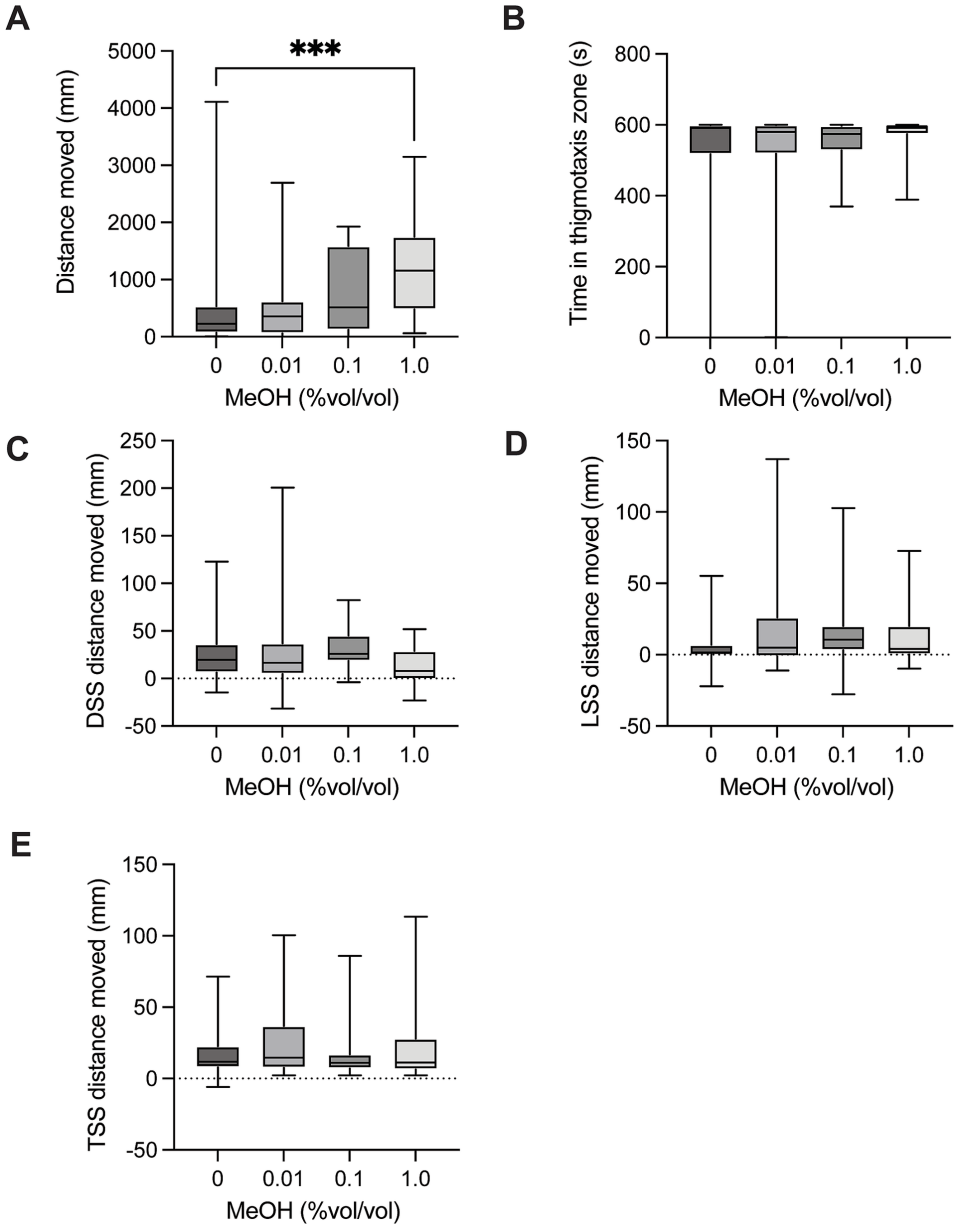
Behavioural responses to methanol dosing. **(A)** Total distance moved in the 10-min spontaneous swim test. There was a significant increase in distance moved for control vs. 1.0 (*p* = 0.0008). **(B)** Total time in the outer thigmotaxis zone during the spontaneous swim test. **(C)** Change in distance moved following the dark startle stimuli (5 s after DSS – 5 s before DSS). **(D)** Change in distance moved following the light startle stimuli (5 s after LSS – 5 s before LSS). **(E)** Change in distance moved following the tap startle stimuli (5 s after 1st TSS – 5 s before 1st TSS). Boxes represent the 25th and 75th percentiles, and whiskers represent the smallest and largest values. The line in the boxes is plotted at the median. Asterix indicate a significant difference (*** = *p* < 0.001).

There was a significant difference in distance moved between control larvae and larvae exposed to methanol [H(3) = 15.12, *p* = 0.0017, Figure 3A] in the spontaneous movement test. *Post hoc* multiple comparison testing showed a significant increase in distance moved for control vs. 1.0% (*p* = 0.0008). There was no significant difference in time spent in the thigmotaxis zone between control larvae and groups exposed to methanol [H(3) = 1.559, *p* = 0.6686, Figure 3B] when testing spontaneous movement. There was no significant difference in dark startle response in larvae exposed to methanol compared to control larvae [H(3) = 7.428, *p* = 0.0594, Figure 3C]. There was no significant difference in light startle response between control larvae and groups exposed to methanol [H(3) = 5.292, *p* = 0.1516, Figure 3D]. There was no significant difference in mechanical tap startle response between control larvae and groups exposed to methanol [H(3) = 1.670, *p* = 0.6437, Figure 3E].

### Ethanol

**Figure 4:**
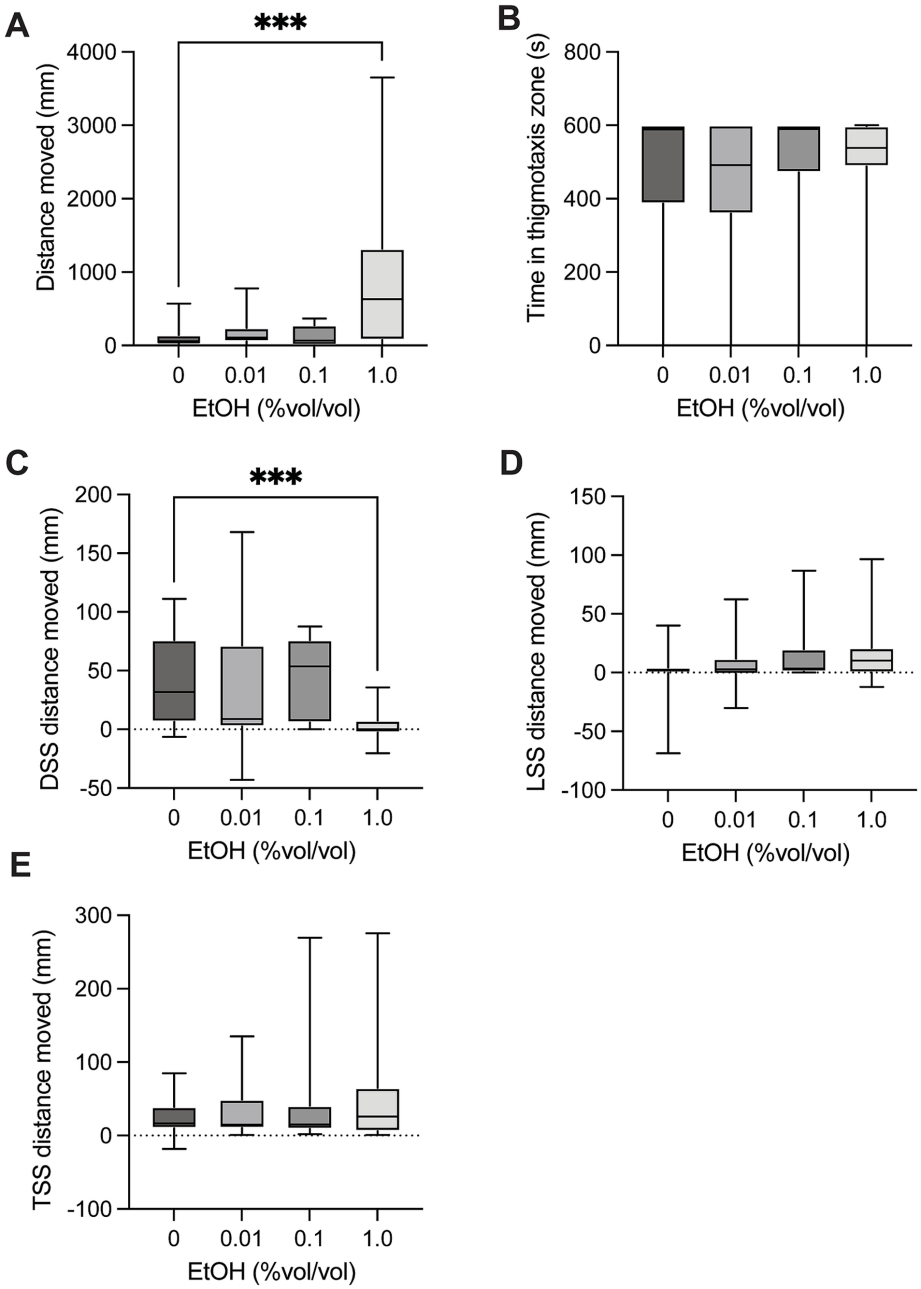
Behavioural responses to ethanol dosing. **(A)** Total distance moved in the 10-min spontaneous swim test. There was a significant increase in distance moved in 1.0 vs. controls (*p* = 0.0004). **(B)** Total time in the outer thigmotaxis zone during the spontaneous swim test. **(C)** Change in distance moved following the dark startle stimuli (5 s after DSS – 5 s before DSS). There was a significant decrease in distance moved for control vs. 1.0 (*p* = 0.0002). **(D)** Change in distance moved following the light startle stimuli (5 s after LSS – 5 s before LSS). **(E)** Change in distance moved following the tap startle stimuli (5 s after 1st TSS – 5 s before 1st TSS). Boxes represent the 25th and 75th percentiles, and whiskers represent the smallest and largest values. The line in the boxes is plotted at the median. Asterix indicate a significant difference (*** = *p* < 0.001).

There was a significant difference in distance moved in control larvae compared to groups exposed to ethanol [H(3) = 17.68, *p* = 0.0005, Figure 4A] in the spontaneous movement test. Multiple comparison testing showed a significant increase in distance moved in with 1.0% ethanol exposure compared to controls (*p* = 0.0004). There was no significant difference in time spent in the thigmotaxis zone between control larvae and groups exposed to ethanol [H(3) = 2.792, *p* = 0.4248, Figure 4B] in the spontaneous movement test. There was a significant difference in dark startle response in control larvae compared to groups exposed to ethanol [H(3) = 23.71, *P* = <0.0001, Figure 4C]. Multiple comparisons showed a significant decrease in dark startle response with 1.0% ethanol compared to control (*p* = 0.0002). There was no significant difference in light startle response between in control larvae and groups exposed to ethanol [H(3) = 4.973, *p* = 0.1738, Figure 4D]. There was no significant difference in mechanical tap startle response in control larvae and groups exposed to ethanol [H(3) = 0.2390, *p* = 0.9711, Figure 4E].

## Discussion

This study describes the impact of acute exposure to three solvents on larval zebrafish behaviour. DMSO, methanol, and ethanol, were tested at 0.01%, 0.1%, and 1.0% vol/vol, which are commonly used with lipophilic compounds. We found DMSO to have no impact on locomotion, zone preference, or startle responses, whereas methanol and ethanol had a dose-dependent impact on locomotion, and we observed a decreased dark startle response with ethanol.

Previous research indicates a significant effect of chronic exposure to DMSO and ethanol at 1% on morphology and mortality from blastula stage to 144 h post fertilization (Chen et al., 2011). With a similar chronic exposure, DMSO, but not methanol at 1% induced behavioural changes (Christou et al., 2020). Shorter 2-day chronic exposures of 0.4% DMSO, however, did not alter behaviour (Jarema et al., 2022). Chronic DMSO exposure caused a dose-dependent increase in the production of stress proteins in larval zebrafish at 5 dpf (Hallare et al., 2004). Here we focused on short term, acute, 30-min exposures comparable to timescales used in many drug screens (Yang et al., 2018; Cassar et al., 2017) and quantified locomotion and responses to three types of startle responses. In this study we found that acute exposure to DMSO at doses up to 1.0% vol/vol does not significantly alter spontaneous locomotion, thigmotaxis behaviour, or startle responses in 5 dpf larval zebrafish. This supports the use of DMSO as a solvent in zebrafish locomotion and startle response assays. DMSO has varying behavioural outcomes based on the developmental stage of the larval zebrafish (Christou et al., 2020). Our study further reinforces that DMSO at doses under 0.55% does not alter the behaviours in larval zebrafish (Christou et al., 2020). DMSO up to 1% seems to be well tolerated in larvae (Hoyberghs et al., 2021) which is consistent with our results. DMSO seems to be less toxic compared to other commonly used solvents, but at higher doses it does become toxic (Kais et al., 2013; Hallare et al., 2006). DMSO seems to be the best option for solvents in acute trials based on our research as it showed no behavioural alterations at any of the doses tested when compared to controls.

Exposure to 1.0% methanol caused a significant increase in spontaneous swimming distance, indicating hyperactivity, while thigmotaxis and startle responses remained unaffected. This suggests that methanol can modulate baseline locomotor activity, potentially through stress or excitatory neural mechanisms, but does not appear to impair sensory-motor integration underlying startle reflexes. In adult zebrafish methanol (0.25, 2.5% vol/vol) exposure for 30 min did not alter distance moved (Hamilton et al., 2021) but in larval zebrafish methanol does impact movement possibly due to increased sensitivity at this early life stage. In another study spontaneous movement in 5 dpf larval zebrafish decreases with higher doses of methanol (3%), although there were also morphological changes and retinal deficits (Fu et al., 2017). Another study found that acute methanol exposure did not alter larval swim speed at 1.5% (Lockwood et al., 2004). When choosing solvents, it is important to consider that methanol seems to have an inconsistent impact on behaviours and in our study, it did alter behaviour in the spontaneous swim test at 1.0%.

Ethanol exposure at 1.0% similarly resulted in increased spontaneous movement; however, it also significantly diminished the dark startle response, indicating a selective attenuation of visual startle sensitivity, but only with the dark stimulus. The absence of effects on light startle and mechanical tap responses suggests that ethanol’s impact may be modality-specific or related to particular neural circuits modulating dark-induced arousal or alertness. In other studies, chronic ethanol exposure in larvae at ≥1.5% impacts larval zebrafish development (Hallare et al., 2006), and 1% ethanol exposure from 1 dpf to 5 dpf causes a decrease in locomotion and a more intense reaction to external stimuli (Du et al., 2020). In another study, ethanol at 1% and 2% increased activity but 4% decreased activity in larval zebrafish in a return to darkness task (MacPhail et al., 2009). Acute ethanol exposure in 7 dpf larvae increased movement speed with a 20-min exposure to a 1.5% dose (Lockwood et al., 2004). Overall, ethanol seems to influence the behaviour and development of larval zebrafish at doses ≥1.0%, which is consistent with our findings.

Results from these experiments highlight the potential impact of ethanol and methanol at 1.0% when used as a solvent with acute dosing experiments. DMSO, in comparison, did not alter locomotion or startle responses. Notably, this timeframe of exposure is not representative of potential effects with repeated, or chronic exposures.

In conclusion, we have shown that acute DMSO up to 1.0% has no significant impact on larval zebrafish behaviours tested in this study. Methanol and ethanol have no impact up to 0.1%, but did show significant behavioural changes at 1.0% causing changes in locomotion. Together, these results highlight the utility of DMSO as a relatively inert solvent in larval zebrafish behavioural research, similar to findings from developmental studies (Hoyberghs et al., 2021) whereas methanol and ethanol require cautious application due to their potential to influence key behavioural endpoints. Furthermore, we only assessed spontaneous movement and startle responses, therefore, caution should be taken with other more complex or sensitive behavioural tests.

## Data Availability

The original contributions presented in the study are included in the article/Supplementary material, further inquiries can be directed to the corresponding author.
